# Exosomes of stem cells: a potential frontier in the treatment of osteoarthritis

**DOI:** 10.1093/pcmedi/pbae032

**Published:** 2024-11-26

**Authors:** Xiaofei Wang, Lei Xu, Zhimin Wu, Linbing Lou, Cunyi Xia, Haixiang Miao, Jihang Dai, Wenyong Fei, Jingcheng Wang

**Affiliations:** The Graduate School, Dalian Medical University, Dalian 116044, China; Department of Orthopedics, Northern Jiangsu People's Hospital Affiliated to Yangzhou University, Yangzhou 225001, China; Department of Orthopedics, Northern Jiangsu People's Hospital Affiliated to Yangzhou University, Yangzhou 225001, China; The Graduate School, Dalian Medical University, Dalian 116044, China; Department of Orthopedics, Northern Jiangsu People's Hospital Affiliated to Yangzhou University, Yangzhou 225001, China; The Graduate School, Dalian Medical University, Dalian 116044, China; Department of Orthopedics, Northern Jiangsu People's Hospital Affiliated to Yangzhou University, Yangzhou 225001, China; Department of Orthopedics, Northern Jiangsu People's Hospital Affiliated to Yangzhou University, Yangzhou 225001, China; Department of Orthopedics, Northern Jiangsu People's Hospital Affiliated to Yangzhou University, Yangzhou 225001, China; Department of Orthopedics, Northern Jiangsu People's Hospital Affiliated to Yangzhou University, Yangzhou 225001, China; Department of Orthopedics, Northern Jiangsu People's Hospital Affiliated to Yangzhou University, Yangzhou 225001, China; Department of Orthopedics, Northern Jiangsu People's Hospital Affiliated to Yangzhou University, Yangzhou 225001, China

**Keywords:** osteoarthritis, cartilage, exosomes, stem cells, regenerative medicine

## Abstract

The aging population has led to a global issue of osteoarthritis (OA), which not only impacts the quality of life for patients but also poses a significant economic burden on society. While biotherapy offers hope for OA treatment, currently available treatments are unable to delay or prevent the onset or progression of OA. Recent studies have shown that as nanoscale bioactive substances that mediate cell communication, exosomes from stem cell sources have led to some breakthroughs in the treatment of OA and have important clinical significance. This paper summarizes the mechanism and function of stem cell exosomes in delaying OA and looks forward to the development prospects and challenges of exosomes.

## Introduction

As the population ages, the prevalence of patients with osteoarthritis (OA) is on the rise, with >240 million people worldwide currently affected [1, 2]. OA is a multifaceted degenerative condition characterized by the deterioration of joint cartilage and inflammation, leading to impaired joint function and limited mobility [3, 4]. The management of OA presents significant clinical, societal, and economic challenges [5]. Being a complex disease influenced by various factors, OA affects both weight-bearing and non-weight-bearing joints and is closely associated with obesity [6]. Given the growing number of individuals affected by OA, the prevention and treatment of this condition have become important medical and social priorities.

The aim of OA treatment is to reduce symptoms, delay joint degeneration, and improve patients′ quality of life. Treatment primarily focuses on preventing cartilage wear and tear. It is divided into conservative treatment and surgical treatment [7]. Surgical treatment is recommended for patients who do not respond well to conservative treatment. In recent years, researchers have been searching for targets to effectively treat OA by inhibiting joint structural damage and achieving long-term improvement [8–10].

All cells release extracellular vesicles (EVs), including exosomes, which are involved in cell-to-cell communication and have various physiological functions [11, 12]. Exosomes play a crucial role in regulating environmental homeostasis, affecting diseases, and promoting processes such as tumor growth, migration, and tissue repair [13–16]. They contain active substances like mRNA, miRNA, DNA, and proteins, and are found in almost all body fluids. Exosomes serve as potential therapeutic targets and have shown potential in stem cell therapy [17, 18]. They participate in intercellular signal transduction, target specific cells, regulate the microenvironment, and promote tissue regeneration. Additionally, exosomes derived from different cell sources are implicated in the development and onset of OA disease [19–21]. The field of exosome research is rapidly advancing and holds significant clinical transformation potential. Therefore, this study aims to investigate the mechanisms and challenges of exosomes in OA, as well as the current state of research in this area.

## Pathogenesis of OA

**Figure 1.**
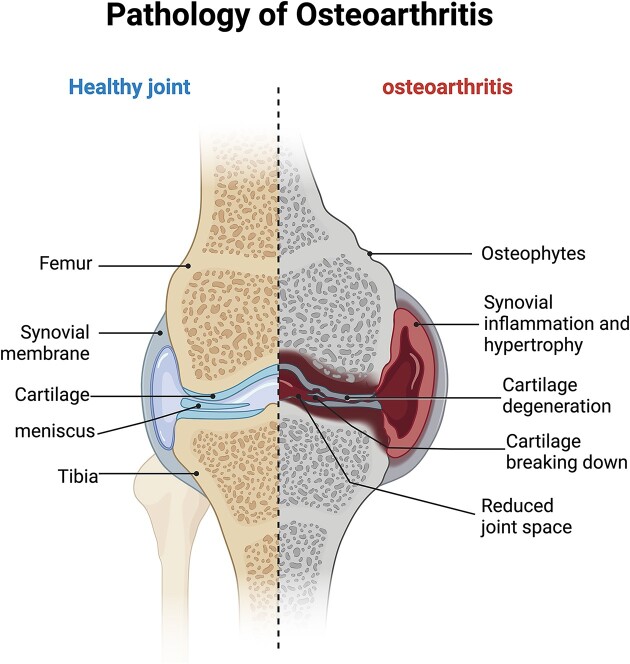
OA is a multifactorial disease affecting the entire joint and its progression involves complex pathological mechanisms. The figure highlights the pathological changes occurring in the joint's cartilage, as well as the alterations in the synovial membrane, subchondral bone, ligaments, and surrounding muscles. (Created with BioRender.com).

OA is a slowly progressing disease that affects the joint tissue, particularly as individuals age. The incidence of OA tends to increase with age. The development of OA is influenced by various factors such as biomechanics, biochemistry, mutations in inflammatory genes, and immunological factors. These factors interact with each other, leading to a cascade of degenerative reactions. As a result, patients with OA experience characteristic changes in the cartilage of their joints, affecting all joint structures [22]. Environmental factors also play a significant role in the pathogenesis of OA, including obesity, metabolic syndrome, dietary changes, and lack of exercise [23]. OA is a multifactorial total joint disease, characterized by the alteration of joint cartilage through complex pathological mechanisms. In addition to affecting the cartilage, OA also impacts the synovial, subchondral, ligament, and muscle tissues surrounding the joint. This complex disease involves inflammation, metabolic disorders, and fibrosis (Fig. 1) [24]. The maintenance of joint cartilage primarily relies on cartilage cells and the extracellular matrix (ECM). An important factor in the development and progression of OA is the increased decomposition metabolism in the articular cartilage ECM [25]. The main proteins in cartilage ECM are type II collagen and aggregated proteoglycans, and the synthesis of ECM forms the foundation for maintaining joint cartilage function [26].

The synovial membrane, which consists of synovial macrophages and synovial fibroblasts, is responsible for secreting joint fluids that lubricate cartilage [27, 28]. In patients with advanced knee OA, apoptotic cells accumulate in the synovial membrane, which can disrupt the important homeostatic function of synovial macrophages and result in the buildup of apoptotic cells [29]. Synovial inflammation in synovial cells can be mediated by mitochondrial dysfunction and damage-related molecular patterns [30]. A number of different tissues or cells communicate with exosomes thereby maintaining the homeostasis of the intra-articular environment or mediating the development of OA [31].

Several pro-inflammatory cytokines play a significant role in the development of OA. Cytokines that are found at elevated levels in OA joint tissues include interleukin (IL)-1β, IL-6, tumor necrosis factor (TNF)-α, monocyte chemotactic protein (MCP)-1, vascular endothelial growth factor (VEGF), and interferon-inducible protein (IP)-10. These pro-inflammatory factors inhibit matrix synthesis by stimulating matrix degradation enzymes, thereby promoting cartilage degradation, which leads to progressive joint destruction and remodeling [32, 33]. Matrix degradation enzymes identified in OA joints include aggrecanase and collagenases, both of which are members of the matrix metalloproteinase (MMP) family, as well as various serine and cysteine proteases. The degradation of the matrix in early OA may be attributed to MMP-3 and aggrecanase, particularly the a disintegrin and metalloproteinase with thrombospondin motifs 5 (ADAMTS-5). This degradation of aggregated proteoglycans is followed by increased collagen activity [34]. Additionally, certain growth factors typically stimulate the production of both matrix and pro-inflammatory factors. For instance, transforming growth factor-β (TGF-β) and bone morphogenetic protein 2 (BMP-2) can promote osteoblastic formation and lead to subchondral sclerosis. These pro-inflammatory mediators and anabolic factors are produced locally by cells within the affected tissues, including chondrocytes in the synovial membrane, synovial fibroblasts, immune cells, inflammatory cells in the perijoint fat, and cells in the bone [35]. Consequently, certain cytokines and growth factors are implicated in the pathogenesis of OA, suggesting that they may represent potential therapeutic targets for delaying the progression of the disease.

## Exosomal therapy

Exosomes, as a therapeutic hotspot, have shown great potential in recent years in the field of joint disease progression. They participate in disease physiology and pathology processes by regulating intercellular communication [36]. The specific role and mechanism of exosomes in OA differ from other biological treatments. Exosome treatment of OA aims to protect chondrocytes from excessive death, reduce inflammation, maintain cartilage matrix metabolism, and regulate angiogenesis and subchondral bone remodeling [37].

### Characteristics, separation, and identification of exosomes

Exosomes are present in all biological fluids and are secreted by all cells. They have the potential to track disease progression [38, 39]. The study of exosomes initially focused on clotting. In the 1940s, Chargaff and West conducted research on blood clotting in New York and discovered a ‘granular part’ that settled at 31000 g with high clotting potential [40]. In the 1980s and 1990s, this substance was identified as a biological entity with enzymatic and functional potential [41]. The term ‘exosome’ was first mentioned in four biomedical articles published in PNAS between 1970 and 1973 [42–45]. These articles described the transfer of transformed DNA fragments between Drosophila or Phlebocytes cells. However, the association of DNA with the lipid bilayer was not mentioned in the literature, so they cannot be easily explained as an early description of EVs from the exosomes studied [46]. Since the late 1980s, exosomes have been consistently referred to as EVs originating in the intracellular system [47–49]. The field of exosomes has rapidly expanded since the earlier research on electron microscopy and biochemistry in the 1940s–80s. Numerous studies have confirmed the findings of the early pioneers in this field, indicating that exosomes play a crucial role in intercellular communication.

The potential for exosomal transformation is being explored for its ability to diagnose, provide prognosis, and treat diseases. Researchers are particularly interested in understanding the regulation of exosomes in various biological processes and diseases [50, 51]. Exosome-based therapy offers advantages such as anti-aging and anti-inflammatory effects, as well as a lower risk of tumor formation and immune rejection compared to stem cell-based therapy. This provides hope for ‘cell-free’ tissue regeneration. In recent years, there has been a significant increase in the study of osteopathic degenerative diseases, with exosomes expected to contribute new therapeutic ideas for the treatment of patients with OA in the future [52].

### Exosomal characteristics

**Figure 2.**
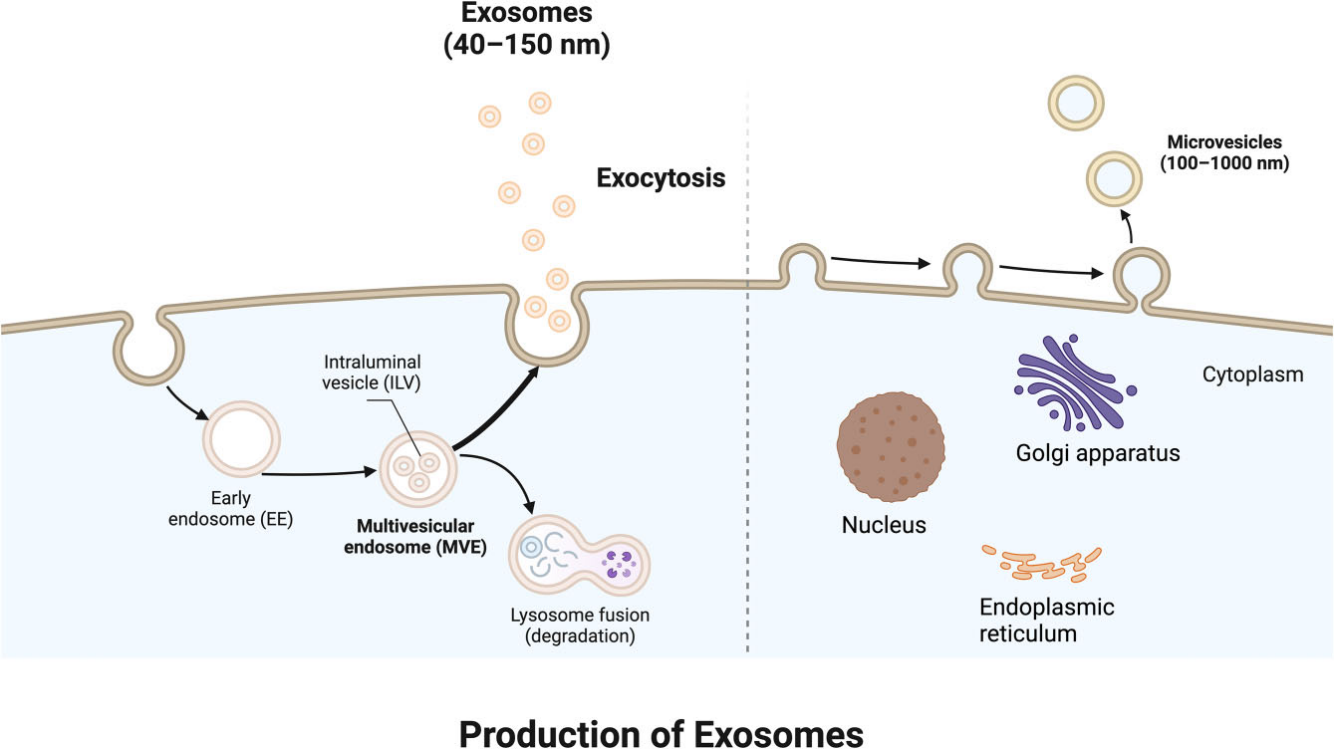
The mechanism of exosome secretion. Exosomes are formed through inward budding of the cell membrane, resulting in the formation of endosomes. These endosomes are then produced through the MVE pathway and subsequently fuse with the cell membrane, leading to exocytosis. The term ‘exosome’ is used to describe these vesicles as they are released into the extracellular environment. (Created with BioRender.com).

EVs are small vesicles that are released from cells into the extracellular space. They consist of various types of vesicles that differ in terms of their biogenesis pathway, size, and composition. EVs are characterized by lipid bilayers and cannot be replicated. They do not contain functional nuclei [53]. The current definition of an EV subtype, called an exosome, is a small EV 30–150 nm in size, produced by the multivesicular endosome pathway [54]. Multivesicular bodies undergo maturation from the endosome as their membranes bud inward in the cavity of the tube, forming vesicles. When these vesicles are released into the extracellular environment, they are referred to as exosomes (Fig. 2) [49, 55].

Exosomes play a crucial role in various physiological and pathological processes, including antigen presentation, regulation of tumor growth and migration, and tissue repair. They also offer unique advantages in early disease diagnosis and targeted drug treatment [56, 57]. Intercellular communication through exosomes is observed in all organisms, ranging from bacteria to plants and animals [58–60]. These exosomes transport proteins, lipids, and RNA, facilitating communication between different cell types and influencing both normal and pathological conditions [61].

### Exosome separation and identification

With the rapid development of biotechnology, exosomes have gained significant research value and prospects. Consequently, it becomes crucial to isolate high-purity exosomes from various biological fluids. Several methods have been developed for exosome separation, taking into account factors such as size, shape, density, and surface protein of the exosome [62]. These methods include high-speed centrifugation, ultrafiltration, immunophilic capture, charge-neutralized polymer precipitation, dimensional resistive chromatography, and microfluidic techniques [63]. Among these, ultracentrifugal separation is considered the classical method, which can be further categorized into density gradient ultracentrifugal separation and differential ultracentrifugal separation. Differential ultracentrifugation, also known as simple ultracentrifugation or precipitation, is one of the commonly employed techniques for exosome separation.

The principle of differential ultra-centrifugation is based on the separation of different extracellular components of fluid samples using centrifugal forces, which separates them sequentially according to their density, size, and shape [64, 65]. Previous studies have extensively used differential ultracentrifugation to isolate exosomes from various sources, including cell media, serum, saliva, urine, and cerebrospinal fluid [66–70]. However, these methods still have limitations such as being time-consuming, requiring immunomagnetic bead capture, and being costly [71]. Further refinement is needed to enhance the study and application of exosomes, considering the advantages and disadvantages of these methods.

Validation of exosomes after separation is commonly assessed using various identification techniques. These techniques include transmission electron microscopy (TEM), nanoparticle tracking analysis (NTA), and size distribution and shape characterization of isolated exosomes [72, 73]. TEM technology is well-established and has been extensively used in exosome studies, providing evidence for the presence of vesicular structures [74]. While standard light scattering NTA does not provide information about the biochemical composition or cellular origin of vesicles, fluorescent labeling can be employed for vesicle analysis [75]. Furthermore, western blot (WB) identification of exosome surface markers such as CD9, CD63, CD81, HSP70, and TSG101 is also a commonly used method for exosome identification [76, 77].

However, these methods possess both advantages and disadvantages. For instance, TEM can effectively observe the morphological structure of exosomes and provide information on particle size distribution. However, due to the complexity of TEM operations and the high demands of sample preparation, it is not suitable for the rapid measurement of large numbers of samples. NTA can detect the size and concentration of exosomes, offering high detection speed and resolution. Nevertheless, its operation is intricate, making it challenging to distinguish between contaminated proteins and exosomes, and its results may be influenced by the quality of the camera used. WB is a commonly employed method for exosome detection, with the advantage of mature technology that allows for qualitative and quantitative analysis of marked proteins. However, WB has notable drawbacks, including a complex and time-consuming identification process, variability in the detection of marker proteins based on cell type, and its unsuitability for detecting exosome marker proteins in biofluids. Flow cytometry can analyze the size of exosomes by targeting them with specific antibodies or fluorescent dyes. Additionally, other techniques such as scanning electron microscopy, atomic force microscopy, adjustable resistance pulse sensing, dynamic light scattering, resistance pulse sensing, enzyme-linked Immunosorbent assay, fluorescence activated cell sorting, as well as microfluidic and electrochemical biosensors, can also be employed to detect exosomes [78]. To ensure the reliability of exosome test results, it is often necessary to utilize multiple methods in conjunction with each other to evaluate the extracted exosomes.

## The mechanism of OA treatment with exosomes

The disease process of OA is a complex one, involving various factors such as cartilage damage, inflammation of the synovial membrane, degeneration of ligaments and synovial membranes, remodeling of the inferior cartilage bone, and changes in the structure of the joint capsule, surrounding muscles, nerves, and local fat pads [79]. Researchers are continuously exploring the underlying pathological mechanisms of OA and developing new strategies to inhibit its progression. Hyperchondrocyte death, ECM degradation, synovial inflammation, neovascularization, nerve invasion, and subchondral remodeling are common pathological manifestations observed during the development of OA [80–82].

**Figure 3.**
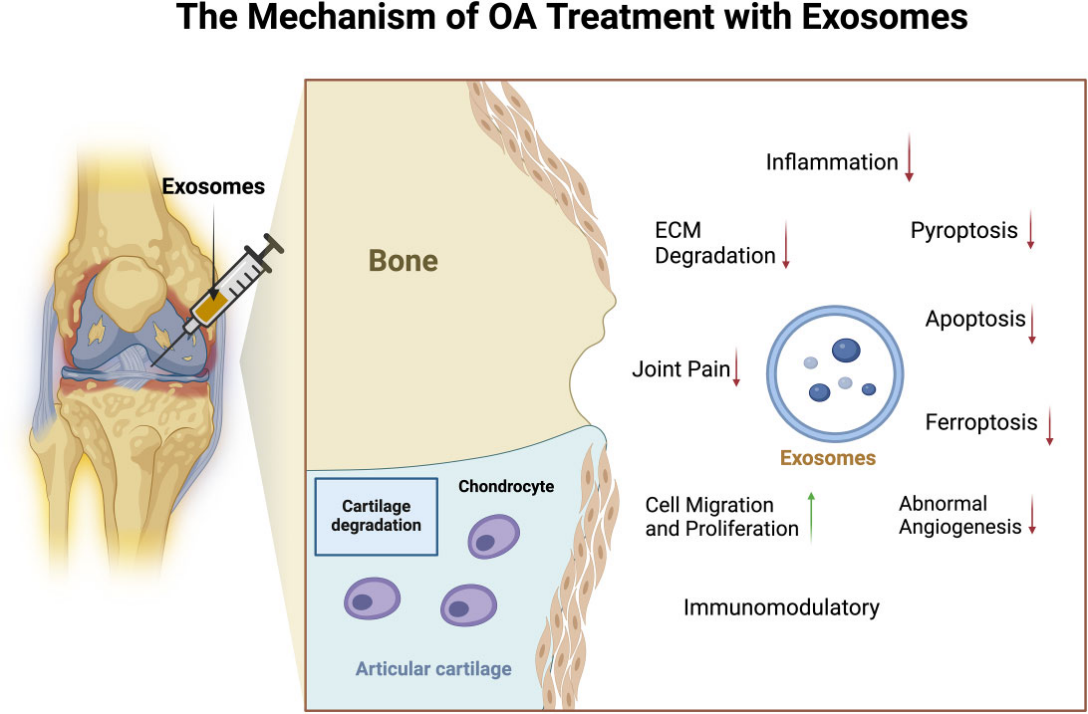
Exosomes play a crucial role in the physiological and pathological processes of OA by regulating intercellular communication. They achieve this by reducing the inflammatory response, influencing the ECM, safeguarding chondrocytes against excessive cell death, regulating the immune system, inhibiting abnormal blood vessel growth, promoting cell migration and proliferation, and alleviating OA pain symptoms. (Created with BioRender.com).

These pathological changes interact to promote the occurrence of OA. While some exosomes of cellular origin (stem cells, chondrocytes, etc.) may have a positive effect on OA, there are others (immune cells, inflamed synoviocytes, diseased subchondral osteoblasts, etc.) that have a negative effect and contribute to the persistent pathogenesis of OA. The exact mechanism of action of exosomes of regenerative or reparative cell origin in the treatment of OA is not yet fully understood. However, numerous studies have demonstrated that exosomes can slow down the progression of OA by inhibiting pathological responses through molecular communication between chondrocytes. This includes reducing the inflammatory response, regulating ECM, protecting chondrocytes from excessive cell death, immune regulation, inhibiting abnormal angiogenesis, promoting cell migration and proliferation, and improving symptoms of OA pain (Fig. 3).

### Reducing the inflammatory response

During the progression of OA, cartilage degenerates as a result of inflammation. This inflammation leads to pathological changes in the cartilage of the joints, triggering the secretion of inflammatory factors. These factors then worsen the damage to the cartilage tissue through signaling pathways, ultimately impacting the OA process. Exosomes have been found to play a crucial role in the biological progression of OA disease [83]. Researchers have identified T lymphocytes and macrophages as the primary inflammatory cells involved in OA [84]. Activation of macrophages results in the release of pro-inflammatory factors, while fibroblast-like synoviocytes and chondrocytes, stimulated by these pro-inflammatory factors, contribute to the degradation of the ECM [85]. Synovial macrophages release pro-inflammatory cytokines, growth factors, and enzymes in response to irritation during inflammatory conditions. Macrophages play a role in stimulating angiogenesis, recruiting leukocytes and lymphocytes, promoting fibroblast proliferation, and secreting proteases. These pathological processes contribute to joint damage [86]. Additionally, oxidative stress activates pro-inflammatory pathways and triggers inflammatory responses and chondrocyte aging, which accelerate the degradation of cartilage ECM during the onset of OA [87–89]. Inflammatory molecules, such as pro-inflammatory cytokines, secreted by OA are crucial mediators in the disruptive processes involved in OA pathophysiology. Specifically, IL-1β and TNF play a significant role in controlling the degeneration of the cartilage matrix in the joints, leading to cartilage destruction and local joint inflammation. Therefore, they are the primary targets of treatment strategies [90, 91].

MicroRNA (miRNA) is a regulator of cellular processes, and miRNA (miR) in exosomes plays a crucial role in intercellular signaling. Kim *et al*. discovered that bone marrow-mesenchymal stem cell (BM-MSC) exosomes treated with IL-1β and TNF-α exhibited significantly enhanced anti-inflammatory activity in SW982 cells of OA. Moreover, the MSC exosome triggered by IL-1β operates through the inhibition of miRNA, such as miR-147b, via the NF-κB pathway [92]. Qiu *et al*. reported that exosomes derived from human synovial-MSCs (HS-MSCs) and carrying miR-129–5p effectively reduced the inflammatory response and apoptosis of chondrocytes after OA treatment. Conversely, the absence of miR-129–5p in HS-MSC-exosomes (HS-MSC-Exos) intensified IL-1β-mediated chondrocyte inflammatory response and apoptosis [93]. In another study, Chang *et al*. demonstrated that exosomes derived from low-oxygen cultured adipose-derived stem cells (ADSCs) alleviate inflammation and slow down the progression of OA by reducing inflammatory cytokines [94]. Jin *et al*. injected miR-9–5p-containing exosomes derived from BM-MSCs into rat OA models to evaluate the levels of inflammatory factors (IL-1, IL-6, TNF-α, and CRP) and the oxidative stress damage index. The results showed that exosomes containing miR-9–5p were able to reduce inflammation [95]. Furthermore, some researchers discovered that miRNA delivered by exosomes from rat synovial fibroblast cells (SFCs) inhibits the expression of IL-1β, IL-6, and TNF-α, thereby improving chondrocyte inflammation and cartilage tissue degeneration [96, 97]. Zhang *et al*. found that exosomes derived from synovial MSCs (SMSC) overexpressing miR-212–5p can effectively inhibit chondrocyte degeneration and inflammation by targeting ELF3 [98]. Thus, miRNAs in exosomes can reduce the inflammatory response of chondrocytes through intercellular signalling, thereby alleviating the progression of OA (Table 1).

**Table 1.** Mechanism of inhibition of OA inflammatory response by exosomes.

Source of exosomes	Main target molecule or pathway	Effect	Reference
BM-MSC	miR-147b	Inhibition of inflammatory reaction by suppressing the NF-κB pathway	[92]
HS-MSC	miR-129–5p	Inhibition of IL- 1β mediated chondrocyte inflammatory response	[93]
AD-MSC	miR-147b	Inhibition of inflammatory reaction by suppressing the NF-κB pathway	[94]
BM-MSC	miR-9–5p	Reduced levels of inflammatory factors (IL-1, IL-6, TNF-α, and CRP)	[95]
SFC	miR-126–3p	Inhibition of the expression of IL-1β, IL-6 and TNF-α	[96]
SFC	miR-214–3p	Inhibition of the expression of IL-1β, IL-6 and TNF-α	[97]
SMSC	miR-212-5p	Inhibition of inflammation by targeting ELF3	[98]

### Regulating breakdown and synthesis of ECM

ECM not only provides a physical scaffold for cell embedding, but also regulates various cellular processes, such as growth, migration, differentiation, survival, homeostasis, and morphogenesis [99]. In the context of OA, the destruction of the cartilage ECM and the disruption of homeostasis are of increasing importance. Under normal physiological conditions, the ECM and chondrocytes mutually nourish each other, with a balanced synthesis and degradation of ECM. However, in OA chondrocytes, there is an imbalance where ECM degradation exceeds synthesis, leading to significant reductions in collagen and protein polysaccharides [100]. This ECM damage directly contributes to the worsening of the disease. During the progression of OA, various molecular pathways in the joints malfunction, affecting the metabolic homeostasis of bone ECM and causing structural disruption and deterioration of its biomechanical properties [101].

Xia *et al*. found that miR-125a-5p-enriched BMSCs-Exo could promote the migration of chondrocytes and remodelled ECM through the upregulation of COL II, aggregated proteoglycans, and SOX 9, as well as the downregulation of MMP-13 [102]. Exosomes derived from human embryonic stem cell-induced MSCs (ESC-MSCs) injected into joints reduced cartilage damage and matrix degradation in DMM models by increasing COL II expression and reducing ADAMTS 5 expression [103]. These exosomes play a therapeutic role in OA by balancing the synthesis and degradation of cartilage ECM. Wang *et al*. found that SMSC has the potential to mitigate damage induced by OA. In mouse models, miR-155–5p-Exos promoted the proliferation and migration of OA chondrocytes and enhanced ECM secretion by targeting runt-related transcription factor 2 (Runx 2) [104]. Tao et al. found that exosomes secreted by SMSC after miR-140-5p overexpression can activate Yes-related proteins(YAP) via alternative Wnt signaling pathways, which enhances the proliferation and migration of joint chondrocytes *in vitro* without compromising ECM secretion. In *in vivo* experiments, SMSC-140-Exos successfully prevented OA in rat models [105]. Thus, miRNAs in exosomes can exert a therapeutic effect on OA through intercellular signalling, which in turn balances the synthesis and degradation of cartilage ECM (Table 2).

**Table 2.** Regulating breakdown and synthesis of ECM in OA.

Source of exosomes	Main target molecule or pathway	Effect	Reference
BM-MSC	miR-125a-5p	Upregulated COL II; aggregated proteoglycans and SOX 9 and downregulated MMP-13; reshaped ECM migration	[102]
ESC-MSC	Unknown	Increased COL II and reduced ADAMTS 5 expression; reduced matrix degradation	[103]
SMSC	miR-155-5p	ECM secretion is promoted by targeting Runx 2	[104]
SMSC	miR-140-5p	Enhances the proliferation and migration of chondrocytes without compromising ECM secretion	[105]

### Protection of chondrocytes from excessive death

#### Inhibiting apoptosis

During OA, there is a significant increase in cartilage cell apoptosis, which scontributes to the further degradation of cartilage tissue [106]. Wang *et al*. conducted a study showing that exosomes derived from adipose tissue-derived MSCs (AD-MSCs) can aid in cartilage regeneration by reducing apoptosis and regulating inflammatory activity. Additionally, they found that miR-486–5p-modified exosomes can inhibit endoplasmic reticulum (ER) stress, decrease cartilage cell apoptosis, and promote matrix regeneration [107]. Xu *et al*. demonstrated that curcumin-sensitized AD-MSC-derived exosomes can effectively reduce oxidative stress and chondrocyte apoptosis in OA cartilage. These findings indicate great potential in the recovery of joint cartilage damage in OA patients [108]. Autophagy and apoptosis are closely related, with autophagy being able to inhibit apoptosis and help maintain cellular stability. In most cases, autophagy inhibits apoptosis or increases the stress threshold required to induce apoptosis [109]. Wang *et al*. discovered that activating transcription factor 4 (ATF4) is crucial for chondrocyte proliferation and bone formation. Intra-articular injection of ATF4-OA-Exo partially restores autophagy and inhibits chondrocyte apoptosis [110]. Wu *et al*. found that exosomes derived from the infrapatellar fat pad (IPFP) MSCs significantly enhance autophagy in cartilage cells by inhibiting mTOR, leading to reduced apoptosis and enhanced matrix synthesis to regulate the progression of OA [111].

#### Inhibiting cell pyroptosis and ferroptosis

Pyroptosis is a regulated form of cell death that may be associated with risk factors for OA. It is involved in cartilage degeneration, synovial changes, and OA-induced pain [112]. Pyroptosis is mediated by NLRP3 inflammators and caspase-1 signaling. In a study in rats, inhibiting NLRP3-mediated inflammation relieved the OA process [113, 114]. Xu *et al*. discovered that BMSC-Exos can deliver miR-326 to chondrocytes and cartilage. Targeting HDAC3 and STAT1/NF-κB p65 to inhibit cartilage and pyroptosis improved OA [115].

Ferroptosis is a form of non-apoptotic cell death that relies on iron. It is characterized by the inactivation of glutathione peroxidase 4 and the accumulation of reactive oxygen species [116]. Recent studies have shown that chondrocytes undergo ferroptosis in the presence of inflammation and iron overload, which promotes the development of OA. Furthermore, chondrocyte ferroptosis promoted articular cartilage MMP-13 expression and inhibited collagen II expression [117–119]. Kong *et al*. discovered that exosomes from OA fibroblast-like synoviocytes containing miR-19b-3p promote cartilage ferroptosis and injury by targeting ferroptosis-related factors such as SLC 7A11 [120]. Cheng *et al*. conducted a study using BMSC-Exos and an iron apoptosis inhibitor to intervene in an OA rat model. They found that BMSC-Exos reduced chondrocyte ferroptosis and prevented the progression of OA by disrupting the METTL3–m6A–ACSL4 axis [121]. Thus, exosomes can protect chondrocytes from excessive death by inhibiting forms of cell death such as apoptosis, pyroptosis, and iron death, thereby alleviating the process of OA (Table 3).

**Table 3.** Protection of chondrocytes from different forms of death in OA.

Source of exosomes	Main target molecule or pathway	Effect	Reference
AD-MSC	miR-486-5p	Suppression of ER stress can reduce IL-1β-induced apoptosis	[107]
AD-MSC	Unknown	Inhibition of apoptosis of articular cartilage cells in mice	[108]
OA mouse serum	Unknown	ATF4 overexpression in OA-Exo promotes autophagy of chondrocytes and inhibits their apoptosis	[110]
IPFP-MSC	miR100-5p	Inhibition of mTOR enhances autophagy levels in chondrocytes to regulate apoptosis	[111]
BM-MSC	miR-326	Targeting of HDAC3 and STAT1//NF-κB p65 to inhibit pyroptosis of chondrocytes and cartilage	[115]
BM-MSC	Unknown	Reduced chondrocyte ferroptosis and prevented OA progression via disruption of the METTL3–m6A–ACSL4 axis	[121]

### Immunomodulation

A growing body of research indicates that the immune system plays a role in the progression of OA. Various factors, such as genetics, metabolism, and mechanical stress, can cause damage to the cartilage. This damage results in the release of specific autoantigens, which then trigger an immune response [122]. Studies have shown that there is infiltration of monocytes in the OA synovial tissue, primarily consisting of CD3+ T cells [123, 124]. Macrophages, T cells, and B cells are the key immune cells involved in controlling the inflammatory process and immune response in OA [125]. These immune cells infiltrate the joint tissues and release cytokines and chemokines from different types of cells present in the joints. This leads to the activation of complement systems and the release of cartilage-degradation factors, such as MMP and prostaglandin E2, which further contribute to the damage of joint cartilage [126–130].

Ragni *et al*. discovered that BMSCs secrete several leukocyte chemokines. BMSC interacts with the abundance of activated immune cells in OA tissues and reduces the pro-inflammatory state through the action of different leukocyte subpopulations. [131]. Additionally, exosomes derived from BMSCs regulate the immune response by controlling the activation and differentiation of T cells, inhibiting B cell function, and reducing the release of inflammatory mediators [132]. Zhang *et al*. demonstrated that EVs from BMSC have an immunomodulatory effect on the regenerative immune phenotype. These EVs can decrease the infiltration of M1 macrophages by attracting M2 macrophages to infiltrate OA cartilage defects and synovial membranes, thereby reducing the expression of IL-1β and TNF-α [133]. Similarly, Zheng *et al*. found that exosomes derived from primary chondrocytes contain more immune-related proteins and can prevent the development of OA by restoring mitochondrial dysfunction and promoting a shift in macrophage response towards the M2 phenotype [134]. Li et al. found that EVs from human umbilical cord-MSCs (HU-MSCs) may promote M2 macrophage polarisation by delivering key proteins and modulating the miRNA-mediated PI3K-Akt signalling pathway, which improves immunomodulation [135]. Exosomes play a significant role in the immune response through various pathways, particularly in the regulation of synovial macrophages. In OA, macrophages may polarize to a pro-inflammatory M1 phenotype. Conversely, M2 macrophages can inhibit inflammation in OA and promote cartilage repair by secreting arginase-1 (Arg-1), IL-10 and TGF-β. Exosomes derived from stem cells of diverse origins may alleviate synovitis and mitigate cartilage degeneration by initiating the transcription of functional genes associated with M2 macrophages, thereby promoting their polarization and inhibiting the infiltration of M1 macrophages into the synovium. Thus, targeting macrophage polarization may represent an effective strategy for modulating inflammatory processes to prevent and reduce the progression of OA. Despite these findings, there are still many uncertainties regarding the interaction between MSC-derived EVs and immune cells related to OA, which require further exploration (Table 4).

**Table 4.** Inhibition of OA by immunomodulation.

Source of exosomes	Main target molecule or pathway	Effect	Reference
BM-MSC	Unknown	Regenerating the immune phenotype reduces the infiltration of M1 macrophages	[133]
Primary chondrocyte	Unknown	Restore mitochondrial dysfunction and polarize macrophage response toward an M2 phenotype	[134]
UC-MSC	miR-122–5p, miR-148a-3p, miR-486–5p, miR-let-7a-5p, miR-100–5p	The polarization of M2 macrophages is facilitated by the PI3K-Akt signaling pathway	[135]

### Inhibiting abnormal angiogenesis

Angiogenesis and adequate blood supply are crucial for bone formation, and OA has the potential to disrupt normal angiogenesis [136, 137]. Hypoxia in chondrocytes plays a key role in gene expression related to angiogenesis in cartilage models. Given that hypoxia promotes angiogenesis in various contexts, hypoxia, in the absence of mesenchymal condensation, is generally considered the primary regulatory factor for angiogenesis associated with intramembrane osteogenesis [138]. Inhibition of cartilage osteogenesis is also linked to a significant decrease in vascular invasion [139, 140]. Wang *et al*. conducted a study where they isolated BMSCs and their exosomes from mice. They discovered that modifying exosomes derived from an MSC source with TGF-β1 helps maintain the microstructure of the subchondral bone in OA mice, inhibits abnormal angiogenesis, and provides protection against OA-induced pain and bone loss [141]. Similarly, Zhao *et al*. found that hypoxia-treated exosomes derived from ADSCs (Hypo-ADSC-Exos) can normalize non-coupling bone remodeling and abnormal H-type angiogenesis in the subchondral bone, thereby improving the progression of lumbar facet joint osteoarthritis (LFJOA) [142]. Therefore, inhibiting abnormal angiogenesis in the cartilage membrane may be one of the strategies to relieve the development of OA (Table 5).

**Table 5.** Inhibition of abnormal angiogenesis in OA.

Source of exosomes	Mainly target molecule or pathways	Effect	Reference
BM-MSC	Unknown	Reduced CD31hiEmcnhi vessel activity in the subchondral bone	[141]
Hypo-ADSC	Unknown	Inhibition of abnormal H-vessel formation	[142]

### Promoting cell migration and proliferation

During the early stages of OA, researchers initially targeted cartilage cells for therapeutic intervention due to increased cell proliferation, matrix protein synthesis, and the presence of proteases and cytokines [143]. Exosomes containing growth factors and ECM-related proteins play a crucial role in promoting chondrocyte proliferation and differentiation, as well as regulating collagen fibrosis and matrix synthesis. As OA progresses, chondrocyte migration and proliferation are hindered, but exosomes can still facilitate cell migration and proliferation [144].

Liu *et al*. discovered that lncRNA-KLF 3-AS 1, derived from exosomes of human MSCs (hMSCs), facilitates the proliferation of OA chondrocytes and the expression of cartilage-forming genes through the miR-206/GIT 1 axis. Furthermore, the expression of MMP13 and its upstream regulator, RUNX 2, is suppressed [145]. Additionally, in rat OA models, researchers observed that exosomal KLF 3-AS 1 from hMSCs promotes cartilage repair and chondrocyte proliferation, while inhibiting IL-1β can induce apoptosis of cartilage cells [146].

One study demonstrated that EVs secreted by BMSCs not only promote cartilage formation but also inhibit hypertrophic differentiation of cartilage cells. Other factors in the BMSC secretion group also aid in controlling the proliferation of OA chondrocytes [147]. Proliferation occurred more rapidly with increasing doses of EV, and when a certain dose was reached it was sufficient to induce chondrocyte migration [148]. Nguyen *et al*. discovered that TGF-β-sensitized umbilical cord MSCs (UC-MSCs) can enhance the effect of EVs on OA chondrocyte migration. Furthermore, the distribution of miRNA in EVs is an important factor that may influence chondrocyte proliferation and migration [149]. Additionally, some researchers have found that EVs produced by MSCs can regulate proteins involved in chondrocyte adhesion, migration, and proliferation [150, 151]. Shao *et al*. found that BMSC-derived exosomes pre-treated with parathyroid hormone enhance their therapeutic effect on repairing OA chondrocytes by inhibiting the expression of pro-inflammatory cytokines. Furthermore, they can promote the migration, proliferation, and formation of cartilage matrix in OA chondrocytes [152]. Thus, exosomes derived from stem cells may promote chondrocyte proliferation and differentiation through their secretion of growth factors, ECM-related proteins, and thus OA chondrocytes proliferation and cartilage formation, thereby relieving OA (Table 6).

**Table 6.** Promoting cell migration and proliferation.

Source of exosomes	Main target molecule or pathway	Effect	Reference
HM-MSC	LncRNA-KLF 3-AS 1	Chondrocyte proliferation is facilitated by the miR-206/GIT 1 axis	[145]
HM-MSC	LncRNA-KLF 3-AS 1	Proliferation of chondrocytes was promoted in a rat OA model by KLF 3-AS 1	[146]
BM-MSC	Unknown	Secreted fibroblast growth factor 1 can promote the proliferation of chondrocytes	[147]
UC-MSC	Unknown	TGF-β sensitization promoted cartilage cell migration	[149]
BM-MSC	Unknown	Enhancement of IL-1β induced proliferation and migration of OA chondrocytes.	[152]

### Improvement of OA joint pain symptoms

The symptoms of joint pain in OA are typically caused by damage to the cartilage [153, 154]. While the primary changes in OA occur in the articular cartilage, there are various structural changes observed in the cartilage, bone, and synovial tissue of OA joints [155, 156]. The bone and synovial membranes may stimulate neuronal sensitization, leading to painful sensations during normal activities [157]. Additionally, inflammatory mediators activate and sensitize nociceptors, which is the main cause of pain in OA. In the advanced stages of the disease, chronic pain resulting from bone friction can lead to mobility loss and psychological impairment [158, 159].

Exosomes play a role in the pain process of OA, and therapy with MSC exosomes can alleviate joint pain symptoms and improve joint function [160]. Calcitonin gene-related peptide (CGRP) is typically involved in transmitting nociceptive signals and sensitizing pain in the peripheral nerve and spinal cord. Neuronal injury in the dorsal root ganglia (DRG) is a significant cause of neuropathic pain and pain sensitization [161]. By controlling inflammation and improving cartilage function, it may be possible to relieve pain in OA patients. He *et al*. discovered that therapy with BM-MSC-Exos significantly reduced the increased expression of CGRP and iNOS in DRG tissue of OA rats, leading to a reduction in joint pain. Furthermore, exosomes decrease anabolic factors such as COL2A1 and ACAN, while increasing the breakdown metabolic factors such as MMP13 and ADAMTS5. These mechanisms promote the repair of cartilage matrix in the OA model, thereby alleviating pain [162].

Li *et al*. investigated the use of BMSC exosomes in treating LFJOA mice. They discovered that these exosomes could alleviate joint pain by eliminating abnormal CGRP-positive nerves and abnormal H-type angiogenesis in LFJ cartilage [163]. Similarly, Yang *et al*. demonstrated that UC-MSCs could relieve OA joint pain in rats by utilizing exosome LncRNA H19 to regulate advanced OA pain through the miRNA-29a-3p/FOS axis [164]. Furthermore, MSC-derived exosomes were found to restore damaged tissue in temporomandibular joint osteoarthritis (TMJOA) cartilage, effectively treating dysfunction and pain associated with TMJOA [165]. Zhang *et al*. also observed that MSC exosomes repaired TMJOA in rat models, resulting in pain suppression, degeneration reduction, inflammation reduction, enhanced matrix expression, and improved structure of the lower cartilage bone, ultimately aiding in joint restoration and regeneration [166]. Therefore, exosomes secreted by stem cells may relieve joint pain caused by OA by regulating neurons and nociceptors. However, the molecular mechanism of exosome involvement in pain has not yet been fully clarified and further research is needed to fully understand the value of exosomes (Table 7).

**Table 7.** Mechanisms to reduce joint pain symptoms.

Source of exosomes	Main target molecule or pathway	Effect	Reference
BM-MSC	Unknown	Regulating the upregulation of CGRP and iNOS in DRG tissue to reduce inflammatory pain and neuropathological pain in OA rats.	[162]
BM-MSC	Unknown	Elimination of abnormal CGRP-positive nerves and abnormal H-angiogenesis in LFJ subchondral bone to relieve pain.	[163]
UC-MSC	miRNA-29a-3p	Improve the pain and central sensitization of advanced OA through LncRNA H19/microRNA-29a-3p/FOS axis.	[164]

### Other mechanisms

In addition to the aforementioned mechanisms, studies have demonstrated that Wnt 3 is an upregulated molecule following acute cartilage injury, contributing to the long-term regeneration of cartilage [167]. Moreover, exosomes can deliver functional active molecules to joint tissue, providing long-lasting protection. The transforming growth factor TGF-β and BMP are critical regulatory factors in cartilage formation [168, 169]. BMP plays a significant role in protecting cartilage from inflammation or trauma by engaging various receptor combinations to activate distinct intracellular signaling pathways. Furthermore, the loss of BMP-related receptor function leads to diminished internal repair of damaged cartilage. Given that TGF-β is essential for cartilage homeostasis, targeting it may represent a viable treatment option. Studies have indicated that TGF-β3 and BMP-6 can induce pluripotent stem cells to enhance cartilage formation, potentially mitigating the progression of OA. Pre-activating MSCs with TGF-β enhances the therapeutic potential of exosomes in OA. By leveraging the benefits of stem cells, TGF-β3, and BMP-6, it is possible to maintain cartilage homeostasis and delay OA progression. Therefore, administering MSC-derived exosomes as combined vectors of TGF-β3 and BMP-6 may offer a novel approach to preventing OA progression [170]. Additionally, to enhance the targeting and functionality of stem cell-derived exosomes, engineering these exosomes for OA treatment is a promising strategy. The engineering preparation methods for exosomes primarily include parent cell pretreatment, drug carrier optimization, and surface modification. Genetic or phenotypic modifications of parent cells can enhance the exosome's function as a highly targeted drug carrier, thereby improving drug efficacy [171]. Furthermore, the pathophysiological changes in hypochondral bone represent a critical process in the progression of OA, with exosome-mediated hypochondral bone remodeling identified as one of the key therapeutic mechanisms. Some researchers have demonstrated that groups treated with BM-MSC-Exos exhibit higher epiphyseal and subchondral bone volume, along with reduced bone degradation. This effect may be linked to the ability of stem cell exosomes to induce the expression of mature joint cartilage cell markers, such as type II collagen and aggregated proteoglycans, while concurrently decreasing the expression of catabolic markers associated with decomposition metabolism (MMP-13, ADAMTS 5) and inflammation (iNOS) [172]. In another study, researchers utilized an exosome injection model derived from dental pulp stem cells in mouse knee joint OA. Their findings indicated that exosomes from dental pulp stem cells effectively improved abnormal hypochondrial reconstruction, inhibited the development of bone sclerosis and osteoderms, and reduced cartilage degradation and synovial inflammation [173].

## Others treatments for OA

### Traditional treatment of OA

OA is a prevalent degenerative joint disease, with traditional treatment options encompassing both conservative and surgical approaches. Physiotherapy and medication are common conservative treatment modalities that may be effective when applied in the early stages of the disease [174–177]. When conservative treatments prove ineffective, surgical intervention is typically considered. For many patients, surgical treatment can significantly alleviate knee pain, improve joint mobility, and enhance overall quality of life [178]. The most prevalent surgical modalities include minimally invasive arthroscopic surgery and joint replacement, with the choice of procedure tailored to the patient's specific condition [179]. Although arthroscopy is advancing rapidly and becoming increasingly common, some patients may require joint replacement surgery within 2 years following arthroscopic procedures [180, 181]. Furthermore, intraoperative malpractice can potentially damage articular cartilage, contributing to the early onset of OA [182]. Intra-articular injections of medications such as corticosteroids and anesthetics can effectively alleviate symptoms in patients with early OA, providing pain relief and restoring transient joint function; however, the efficacy and safety of long-term use remain uncertain. Additionally, intra-articular drug injections do not reverse the progression of OA by eliminating pathogenic factors or promoting cartilage regenerative repair [183, 184]. Consequently, there is an urgent need to identify and implement more direct and effective treatments to address the growing number of patients affected by OA.

### Biotherapy of OA

The current state of OA research indicates a growing interest in regenerative medicine for treating cartilage regeneration in joints. This approach aims to remodel the joint structure and effectively treat OA [185]. Regenerative methods are designed to address the loss of cartilage matrix by stimulating cartilage formation in endogenous stem cells and matrix metabolism in cartilage cells. This restoration process aims to restore the normal structure and function of damaged joints [186]. In recent years, various biotherapies, such as cell therapy, gene therapy, and biomaterials, have emerged in preclinical or clinical trials. These biotherapies primarily focus on regulating the microenvironment and cellular activity within the joint [187, 188]. Additionally, current OA research emphasizes disease prevention and early treatment, making biologics increasingly attractive due to their ability to intervene in OA diseases and promote cartilage regeneration. Inflammation is now recognized as a key pathophysiological process in OA, and the use of biological agents to address the inflammatory response is a crucial aspect to consider [189].

#### Cell therapy

Cell therapy has emerged as a prominent topic in the medical field in recent years as an alternative treatment for tissue damage. By utilizing various types of cells, it aims to alleviate the pathogenesis of diseases [190]. This therapeutic approach holds great potential in regenerating lost cartilage, combating cartilage degradation, relieving pain, and improving patient mobility [191]. Among the different sources of cells for regenerative medicine, MSCs have shown promise. These cells can be derived from adipose tissue, bone marrow, synovial tissue, and other sources. Currently, the MSCs being studied include BM-MSCs, AD-MSCs, and UC-MSCs [192–195]. Another highly potent tissue engineering therapy for joint cartilage injury is induced pluripotent stem cells (iPSCs). These cells have the ability to differentiate into chondrocytes and can be reprogrammed from somatic cells, offering a wider range of sources and avoiding ethical concerns [196]. iPSCs have the potential to overcome limitations associated with current cellular sources, as they allow for the generation of large numbers of autocytes from small starting groups. Moreover, iPSCs show promise as a viable source of cells for treating cartilage defects and could be directly utilized in clinical applications [197]. Researchers have also observed self-renewal activity in iPSCs, enabling the production of homogeneous iPSC-derived cartilage that can be transplanted to an unlimited number of patients. This approach addresses issues related to allogeneic cartilage, such as donor scarcity, risk of disease transmission, and variations in cartilage quality among donors [198]. These cells have the ability to proliferate, differentiate, metabolize, and replenish lost cartilage cells, as well as the ECM, thereby facilitating the repair of damaged joint cartilage and restoration of joint function.

It is important to acknowledge the existing limitations in the current application of cell therapy for the treatment of OA [199]. One significant challenge is the unsustainable cellular and hyaline cartilage phenotype of differentiated chondrocytes [200]. Additionally, the onset of senescence in MSCs adversely impacts their differentiation potential, immunomodulatory capacity, and migratory ability. Even with cryopreservation, studies have shown a decline in viability, colony-forming units, and integrin expression following thawing [201, 202]. With the rapid increase in the number of stem cell studies, there are concerns regarding the safety of stem cell therapy. For example, stem cell injections can potentially lead to cell transformation or premature cell differentiation [203, 204]. Currently, stem cell therapy remains a topic of controversy due to the immune regulatory function of stem cells, which may contribute to the development of tumors [205]. Additionally, there are still unresolved issues related to the risks of infection transmission during cell transplantation, ethical concerns surrounding embryonic stem cells, limitations on the potential of adult stem cell differentiation, and changes in the characteristics of stem cells after *in vitro* culture [206, 207]. Consequently, the development of cell therapy for the treatment of OA will require significant time and effort.

#### Growth factor therapy

Growth factors are believed to have the potential to enhance the healing of cartilage injuries and modify the progression of degenerative OA [208]. These growth factors can stimulate the differentiation of MSCs to produce phenotypes that resemble normal joint cartilage and possess similar biomechanical properties [209]. Platelet-rich plasma (PRP) is an autologous concentrate derived from human platelets, containing a substantial amount of growth factors alongside elevated concentrations of platelets, leukocytes, and fibrin [210]. By directly injecting these growth factors into the affected joint cavity, it is possible to stimulate chondrocyte proliferation and ECM synthesis, potentially delaying or even reversing the progression of degenerative joint disease. Moreover, the growth factors and other bioactive molecules present in PRP may facilitate critical tissue healing and alleviate pain by modulating inflammation, inhibiting chondrocyte apoptosis, and promoting collagen synthesis. This has been shown to result in pain relief and functional improvement in patients with mild-to-moderate knee OA [211–214]. Park *et al*. evaluated the efficacy of intra-articular PRP injections in comparison to hyaluronic acid (HA) injections for the treatment of OA. They observed that patients receiving PRP exhibited high concentrations of growth factors, suggesting that these concentrations could serve as important indicators in future studies investigating the role of PRP in OA treatment [215].

#### Gene therapy

Gene therapy corrects or treats diseases caused by abnormal genes by introducing exogenous genes into target cells. It also provides new insights for the treatment of OA [216]. Advances in OA genetics, genomics, and epigenetics have enhanced our understanding of the complexity of the disease and guided the evaluation of genetic/epigenetic findings for translation and clinical application [217]. In recent years, genome-wide association studies have identified numerous novel genetic risk loci for OA. Epigenetic traits and mediators serve as a mechanistic link between genetic risk factors for OA and the onset or progression of the disease. Furthermore, since epigenetic traits are relatively easy to modulate, they present potential therapeutic targets [218, 219]. Some researchers have demonstrated significant differences between different OA patient populations, for example based on disease severity, affected joint sites, and gender, and have highlighted attractive drug targets [220].

Attur *et al*. conducted a study on the relationship between the single nucleotide polymorphism of the interleukin-1 receptor antagonist (IL-1RN) gene and the radiological severity of symptomatic OA, as well as the risk of developing OA. The study revealed that IL-1RN gene variation can predict the radiological severity and risk of OA in the knee [221]. In recent times, the application of OA genetics in large-scale genome-wide association scans has made significant progress, identifying >100 polymorphic DNA variants associated with OA. These genetic risk variants account for >20% of OA heritability and are primarily located in non-protein coding regions of the genome, suggesting that they function by regulating the expression of the target gene. Although the data from OA genetics studies have not directly led to new treatments, it is worth noting that some OA-related genes encode proteins for which treatments are already available [222]. Further research in osteogenetics has the potential to provide valuable insights into the pathogenesis of OA, and by identifying common molecular mechanisms, genetic understanding may help uncover causal pathways [223]. Consequently, a deeper understanding of the molecular basis of OA subtypes will enhance our knowledge of the molecular processes involved in the development of OA and contribute to improved patient diagnosis, management, and treatment.

## Conclusion and perspectives

The pathology of OA is complex and can be influenced by a variety of factors such as environmental, genetic, metabolic, or mechanical injury. Exosomes are involved in the physiological and pathological processes of OA by regulating intercellular communication and show strong potential for application in the treatment of OA.

Despite all these promising preclinical results, there are currently several issues that hinder the practical application of exosomes as regenerative therapies in clinical OA treatment. Current studies on stem cell-derived exosomes as therapeutic agents for OA are primarily conducted using *in vitro* cellular models or *in vivo* models involving small animals. Their efficacy has been evaluated in fewer studies involving large animals, which may not yield significant clinical effects. Furthermore, the optimal dose and frequency of exosomes required for clinical application remain inadequately defined. Many studies have utilized higher doses or more frequent injections to demonstrate significant improvement in animal models of OA; however, the translation of these findings to human treatment requires further investigation. There is a notable lack of standardization in the methodologies employed across studies regarding the classification of exosomes, isolation techniques, characterization, stem cell sources and growth conditions, selection of OA models, outcome measurement types, and study durations. Consequently, results from different studies often lack reproducibility. Additionally, exosomes used in clinical trials must adhere to good manufacturing practices. Therefore, the development of pharmaceutical products with stable quality, clear and enhanced efficacy, and scalable production of stem cell-derived exosomes, as well as the enhancement of their druggability, are critical clinical translational issues that warrant emphasis and further exploration. Given that the biological effects of exosomes are mediated through their uptake by target cells, it is essential to clarify and control the biodistribution of exosomes for effective clinical therapeutic applications.
